# *BoLA-DRB3* Polymorphism Associated with Bovine Leukemia Virus Infection and Proviral Load in Holstein Cattle in Egypt

**DOI:** 10.3390/pathogens12121451

**Published:** 2023-12-14

**Authors:** Rania Hamada, Samy Metwally, Ryosuke Matsuura, Liushiqi Borjigin, Chieh-Wen Lo, Alsagher O. Ali, Adel E. A. Mohamed, Satoshi Wada, Yoko Aida

**Affiliations:** 1 Viral Infectious Diseases Unit, RIKEN, 2-1 Hirosawa, Wako, Saitama 351-0198, Japan; raniahamada1990@gmail.com (R.H.); samy_gamal@vetmed.dmu.edu.eg (S.M.); matsuura-ryosuke@g.ecc.u-tokyo.ac.jp (R.M.); b-liushiqi@ous.ac.jp (L.B.); rogerwen80@gmail.com (C.-W.L.); 2 Department of Pathology and Clinical Pathology, Faculty of Veterinary Medicine, Damanhour University, Damanhour City 22511, Egypt; 3 Division of Infectious Diseases, Department of Animal Medicine, Faculty of Veterinary Medicine, Damanhour University, Damanhour City 22511, Egypt; 4 Laboratory of Global Infectious Diseases Control Science, Graduate School of Agricultural and Life Sciences, The University of Tokyo, 1-1-1 Yayoi, Bunkyo-ku, Tokyo 113-8657, Japan; 5 Department of Animal Medicine, Faculty of Veterinary Medicine, South Valley University, Qena City 83523, Egypt; alsagher.ali@vet.svu.edu.eg (A.O.A.); adel.mohamed@vet.svu.edu.eg (A.E.A.M.); 6 Photonics Control Technology Team, RIKEN Center for Advanced Photonics, 2-1 Hirosawa, Wako, Saitama 351-0198, Japan; swada@riken.jp

**Keywords:** BLV, Holstein cattle, proviral load, BLV infection, *BoLA-DRB3*, polymorphism, association study

## Abstract

Bovine leukemia virus (BLV) is the etiological agent of enzootic bovine leukosis, the most prevalent neoplastic disease of cattle worldwide. The immune response to BLV and disease susceptibility and resistance in cattle are strongly correlated with the bovine leukocyte antigen (*BoLA*)-*DRB3* allelic polymorphism. BLV infection continues to spread in Egypt, in part because the relationships between BLV infection, proviral load in Egypt, and *BoLA-DRB3* polymorphism are unknown. Here, we identified 18 previously reported alleles in 121 Holstein cows using a polymerase chain reaction sequence-based typing method. Furthermore, *BoLA-DRB3* gene polymorphisms in these animals were investigated for their influence on viral infection. BoLA-DRB3*015:01 and BoLA-DRB3*010:01 were identified as susceptible and resistant alleles, respectively, for BLV infection in the tested Holsteins. In addition, BoLA-DRB3*012:01 was associated with low PVL in previous reports but high PVL in Holstein cattle in Egypt. This study is the first to demonstrate that the *BoLA-DRB3* polymorphism confers resistance and susceptibility to PVL and infections of BLV in Holstein cattle in Egypt. Our results can be useful for the disease control and eradication of BLV through genetic selection.

## 1. Introduction

Enzootic bovine leukosis (EBL), the most common neoplastic disease in cattle worldwide, is caused by bovine leukemia virus (BLV), which belongs to the *Retroviridae* family [1,2]. Most BLV-infected cattle show no clinical symptoms, and 30% of infected cattle develop polyclonal non-neoplastic B-cell lymphocytosis, while malignant B-cell lymphomas occur in only 1% to 5% of BLV-infected cattle that are 5 to 10 years of age [1,2]. There is considerable economic loss commonly caused by BLV infection, such as premature death of cattle by lymphomas [3], whole carcass disposal at slaughter [4], low milk yield [5,6,7,8,9], weakness of immunity [10,11], affected reproductive capacity [12,13], and affected longevity [5,7,14]. The DNA copies of the BLV RNA genome integrate into the host genome as a provirus, and the BLV provirus remains integrated in the cellular genome, even in the absence of detectable BLV antibodies, inducing lifelong infection [15]. Therefore, BLV is likely to be spread via blood containing infected cells, and it can occur through bloodsucking insects, needle sharing, gloves for rectal examinations, dehorning, tattooing, and castration [16]. The proviral load (PVL), which is the number of copies of a provirus, is related to BLV-associated disease prediction [17,18,19]; BLV infectivity [20,21]; number of lymphocytes [22]; viral biokinetics [23]; shedding of the virus into saliva, nasal mucus [24], and milk [25,26]; and distribution of the BLV in the whole body [27]. Thus, PVL is an important diagnostic index for estimating BLV transmission risk [28].

Previous studies have identified polymorphisms within the bovine major histocompatibility complex (MHC) as host factors strongly associated with BLV PVL [29,30,31]. In cattle, the MHC system is termed the bovine leukocyte antigen (BoLA). *BoLA* has been indexed as a marker for illness and immunological traits in cattle. In particular, *BoLA-DRB3* is the most polymorphic and is a highly expressed class II locus [32]. The Immuno Polymorphism Database (IPD)-MHC database (https://www.ebi.ac.uk/ipd/mhc/group/BoLA/ (accessed on 1 January 2023)) currently lists 384 *DRB3* alleles. This allelic information will assist with continued attempts to catalogue the frequencies of *BoLA* alleles according to breed and area and will be crucial for examining the connection between MHC and disease [32]. Many investigations have revealed a link between one or more *BoLA-DRB3* alleles and susceptibility or resistance to a range of infectious diseases in cattle, including mastitis [33,34,35,36], dermatophilosis [37], tick-borne disease [38,39], foot-and-mouth disease [40], and bovine herpesvirus 1 [41]. Additionally, *BoLA* allele associations with aspects related to milk production, growth, and fertility have been observed [34,42,43]. It has also been noted that *BoLA-DRB3* polymorphisms affect vaccine efficiency, such as vaccines for foot-and-mouth disease and *Theileria parva* [44,45]. Moreover, we previously identified that the *BoLA-DRB3* polymorphism in BLV-infected cattle is associated with PVL, infectivity in blood, development of lymphoma, and in utero infection of calves [21,29,30,31,32,46,47,48,49].

Through the continuing trade of breeding cattle in many countries, BLV has spread worldwide [50]. Milk production from foreign breeds of cattle, particularly Holstein, Holstein Friesian, and Simmental cattle, is a major source of revenue for Egypt’s dairy industry. The first EBL outbreak in Egypt occurred in 1997 [51]. Several serological studies have detected BLV infections among dairy cattle in Egypt from different regions, such as Kafr El Sheikh, Alexandria, and Menofia, with infection rates of 15.83% [52], and 20.8% in cows in Kafr El Sheikh, Qalyubia, and Menofia [53]. Our recent investigation of the distribution of BLV provirus revealed a 25–28% BLV infection rate for healthy male beef cattle in three slaughterhouses located in the central and southern regions of Egypt [54] and 21.5% for dairy cattle in five provinces located in the northern, central, and southern regions of Egypt [55]. According to our earlier reports, phylogenetic analysis of the partial env-gp51 sequence of the isolated BLV strains showed that BLV genotypes 1 and 4 coexist in dairy and beef cattle in Egypt from the 12 globally detected genotypes [54,55]. Estimation of BLV PVL among dairy cattle in five provinces in Egypt [55] demonstrated that the BLV PVL level varied among different regions of Egypt with PVL ranging from 7 to 98,725 copies/10^5^ cells as calculated by the BLV-CoCoMo quantitative polymerase chain reaction (qPCR)-2 method for the quantitative measurement of PVL [17,56]. However, no studies have examined the relationship between BLV infection or PVL and *BoLA-DRB3* polymorphism in Egypt.

Allelic markers of host genetic variants of *BoLA-DRB3* could be the basis of a strategy for managing BLV in Japan in the current absence of applicable vaccinations or effective treatments [57]. Thus, genetic selection is useful for disease control and the eradication of BLV. It is important to understand the genetic characteristics of *BoLA-DRB3* alleles in different cattle breeds in Egypt. However, the diversity of the *DRB3* gene in cattle in Egypt remains unclear. In addition, no studies have examined the relationship between the *BoLA-DRB3* polymorphism and BLV infection in Egypt. Moreover, the *BoLA-DRB3* polymorphism is associated with PVL levels in the blood of infected cattle, as the main indicator of BLV transmission, and has not yet been studied in Egypt.

In this study, we examined the distribution of *BoLA-DRB3* alleles in Holstein cattle in Egypt and their relationship with BLV PVL in infected individuals.

## 2. Materials and Methods

### 2.1. Animals and Sampling

A total of 121 lactating imported Holstein females 5–10 years of age from three dairy farms were used in this study. Detailed information about the animals/farms analyzed is provided in Table S1. Whole blood samples were collected from animals using a coccygeal venipuncture procedure. Collected blood samples were transferred to sterile glass tubes containing K2 EDTA anticoagulant. Then, the blood samples were transported immediately (within 1–4 h) to the laboratory and kept at 4 °C until the DNA extraction was carried out.

### 2.2. Isolation of Genomic DNA from Whole Blood

The blood samples collected from each farm were subjected to genomic DNA extraction on the same day as collection or within 24 h after. Genomic DNA was extracted from 300 µL whole blood using the Wizard Genomic DNA Purification Kit (Promega, Madison, WI, USA), according to the manufacturer’s instructions. The concentration of the isolated DNA samples was determined using a NanoDrop One Spectrophotometer (Thermo Fisher Scientific, Waltham, MA, USA). For PCR tests, DNA samples were diluted in water free of nuclease to a final concentration of 30 ng/µL.

### 2.3. Determination of BLV Infection and Quantification of BLV PVL Using the BLV-CoCoMo-qPCR-2 Assay

BLV PVLs were quantified using the BLV-CoCoMo-qPCR-2 assay as described previously [17,56]. In brief, the degenerate primer pairs CoCoMo FRW and CoCoMo REV and a 15 bp 6-carboxyfluorescein (FAM)-labeled LTR probe were used to amplify a 183 bp sequence of the BLV LTR regions (Table S2). A 151 bp sequence of *BoLA-DRA* was amplified using the primer pairs DRA-FW and DRA-RW and a FAM-labeled DRA probe (Table S2). The *DRA* gene was used as an internal control to normalize the viral genomic DNA level within the host cellular genome. BLV Plasmid DNA/Dilution Solution was used as a standard, with BLV positive control/negative control (Nippon Gene, Tokyo, Japan), in a reaction mixture containing THUNDERBIRD Probe qPCR Mix (Toyobo, Tokyo, Japan) following the manufacturer’s instructions. PCR cycles were performed by using a Roche Light Cycler^®^ 480 Instrument II plate-based real-time PCR amplification system according to the following program: 1 min incubation at 95 °C followed by 45 cycles of 15 s at 95 °C and 1 min at 60 °C. Finally, PVL was expressed as the proviral copy number in 100,000 PBMCs and calculated according to the following formula: (number of BLV *LTR* copies/number of *BoLA-DRA* copies) × 10^5^ cells.

BLV infection was determined using the BLV-CoCoMo-qPCR-2 assay as follows: cattle tested by BLV-CoCoMo-qPCR were negative (−) if the calculated PVL = 0 and positive (+) if the calculated PVL was ≥1, as described previously [58].

### 2.4. Genotyping of BoLA-DRB3

A PCR-sequencing-based typing (SBT) technique was used to type *BoLA-DRB3* alleles using the PCR primers DRB3FRW and DRB3REV to amplify exon 2 of the *BoLA-DRB3* gene as shown in Table S2 [59]. PCR amplification was performed using an r-Taq DNA Polymerase kit (Toyobo). The reaction mixture was prepared following the manufacturer’s instructions. The DNA template was used at a concentration of 30 ng/µL, while the DNA of the KU-1 cell line and nuclease-free water were used as positive and negative controls, respectively. The thermal cycling profile consisted of the following: initial denaturation for 2 min at 94 °C, 35 cycles of denaturation for 20 s at 94 °C, annealing for 30 s at 57 °C and extension for 60 s at 72 °C, and final extension for 2 min at 72 °C. An amount of 5 µL of the reaction mixture after the PCR reaction was fractionated by electrophoresis on 2% agarose/TAE gel. Then, the gel was stained with ethidium bromide, electrophoresed at 100 V for 30 min, and bands of DNA were visualized under UV light using a 100 bp DNA ladder. The amplified PCR fragments (~300 bp) were purified using an ExoSAP-IT PCR product purification kit (USB Corp., Cleveland, OH, USA) and sequenced using the ABI PRISM BigDye1.1 Terminator Cycle Sequencing Ready Reaction Kit (Applied Biosystems, Foster City, CA, USA) and primer set (DRB3FRW and DRB3REV) for sequencing in both directions. The raw sequence data were analyzed using Assign 400ATF ver. 1.0.2.41 software (Conexio Genomics, Fremantle, WA, Australia) to determine the *BoLA-DRB3* genotype.

### 2.5. Statistical Analysis

Allele frequencies and the number of alleles were determined by direct counting. The association between the *BoLA-DRB3* allele and BLV infection profile was determined based on Fisher’s exact test by comparing the frequency distribution of alleles between BLV provirus-negative and -positive cattle. The alleles associated with the BLV infection profile were estimated based on the odds ratio (OR). The association between the *BoLA-DRB3* allele and BLV proviral load profile was determined based on the Chi-square test by comparing the frequency distribution of alleles between cows with low or high proviral loads (LPVL and HPVL, respectively). Statistical analyses were performed using the GraphPad Prism 7 software (GraphPad Software Inc., La Jolla, CA, USA).

## 3. Results

### 3.1. Genotyping of Holstein Cattle’s BoLA-DRB3

All examined Holstein cows were successfully genotyped for the *BoLA-DRB3* gene using an SBT assay. Eighteen previously reported alleles were found in 121 Holstein cows. Eight of these alleles were found more frequently than 5%. The remaining 10 alleles were found less frequently (<5%) (Table 1). The eight most frequently occurring alleles were DRB3*001:01, DRB3*009:02, DRB3*010:01, DRB3*011:01, DRB3*012:01, DRB3*014:01:01, DRB3*015:01, and DRB3*027:03, with frequencies of 15.70%, 7.44%, 7.44%, 16.94%, 6.61%, 8.68%, 20.25%, and 6.20%, respectively. The frequencies of the remaining 10 alleles, DRB3*002:01, DRB3*005:01, DRB3*006:01, DRB3*007:01, DRB3*007:04, DRB3*008:01, DRB3*013:01, DRB3*016:01, DRB3*017:01, and DRB3*018:01, were 2.89%, 0.41%, 0.83%, 1.65%, 0.83%, 0.83%, 0.41%, 1.24%, 0.83%, and 0.83%, respectively. The results showed that DRB3*015:01 was the most abundant allele (20.25%), with a range of 15.48–25.98% at a 95% confidence interval, whereas DRB3*005:01 and DRB3*013:01 were both the least occurring alleles (0.41%), with a range of 0.02–2.64% at a 95% confidence interval in the tested Holstein cows.

### 3.2. Analysis of the Association between BoLA-DRB3 and BLV Infection in Holstein Cows

First, to determine the association between *BoLA-DRB3* and BLV infection in the investigated Holstein cattle population, we compared *BoLA-DRB3* allelic frequencies between non-infected (*n* = 76) and infected (*n* = 45) Holstein cattle, as summarized in Table 2. Notably, DRB3*015:01 was the most frequent allele in the infected group, and DRB3*011:01 was the most frequent allele in the non-infected group. DRB3*002:01, DRB3*009:02, and DRB3*014:01:01 were more frequent among non-infected Holstein cows than infected cows. Furthermore, DRB3*005:01, DRB3*007:01, DRB3*008:01, DRB3*010:01, DRB3*013:01, and DRB3*016:01 were present in the non-infected group and were absent in the infected group. OR and *p*-values were quantified for each allele using Fisher’s exact test. Alleles with OR > 1 and <1 were categorized as resistant and susceptible, respectively. OR was >1 for DRB3*002:01, DRB3*009:02, DRB3*010:01, DRB3*011:01, and DRB3*014:01:01, but only DRB3*010:01 was significantly (*p* < 0.0001) associated. Therefore, DRB3*010:01 was considered an allele resistant to BLV infection. Fisher’s exact test for allele association indicated a significant association for DRB3*015:01 (*p* < 0.0001) and it was determined to be a susceptible allele for BLV infection in the tested Holsteins.

### 3.3. BLV PVL Estimation in Holstein Cattle and Classification into Three Groups by Categorizing PVL

**Figure 1:**
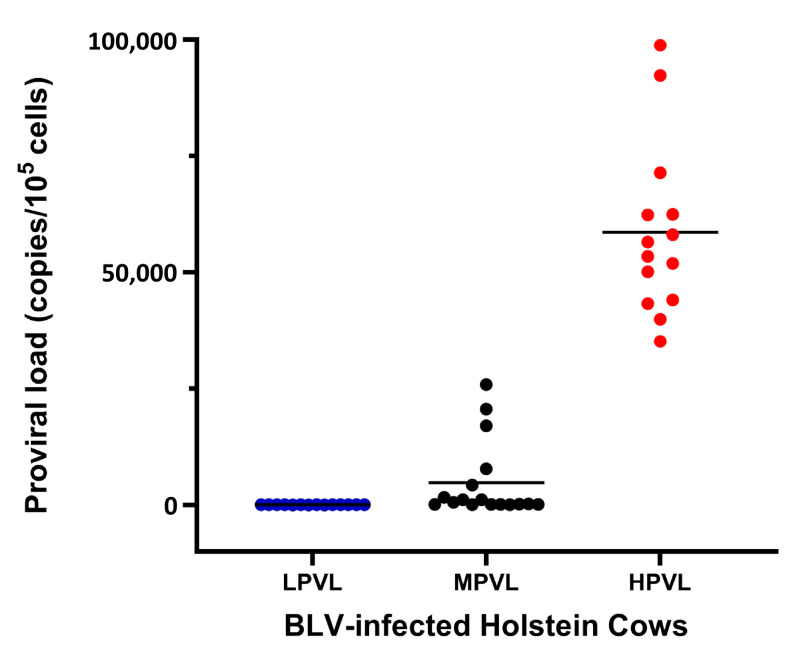
Comparison of proviral load (PVL) in blood from BLV-positive cows. BLV PVLs calculated using CoCoMo-qPCR-2. LPVL: cows with low PVL (34 ≤ PVL ≤ 85 copies per 10^5^ cells), *n* = 14 cows; MPVL: cows with moderate PVL (89 ≤ PVL ≤ 25,858 copies per 10^5^ cells), *n* = 17 cows; HPVL: cows with high PVL (35,138 ≤ PVL ≤ 98,725 copies per 10^5^ cells), *n* = 14 cows. Black bars show the main BLV PVL values.

It was previously reported that cows with a PVL > 10,000, 14,000, and 18,000 copies per 10^5^ cells excreted BLV into milk, nasal mucus, and saliva, respectively [24,25]. In addition, blood PVL was positively associated with the distribution of the BLV provirus in the organs during the early stages of BLV infection [26]. It appears that PVL plays a direct role in the risk of BLV transmission in herds. Cattle with a high PVL are considered a risk for virus transmission on a farm. In the BLV-CoCoMo-qPCR-2 experiment, we amplified the *BoLA-DRA* gene, a single-copy host gene, in tandem with the viral genomic DNA to determine the cell number and proviral load (expressed as the number of copies of the provirus per 100,000 cells). Our findings showed that 45 of the 121 samples tested positive for BLV (37.19%), with varying quantities of viral copies in the blood. We found a wide range of viral copies, from 34 to 98,725 copies per 10^5^ cells, with a mean of 20,027 copies per 10^5^ cells (Figure 1).

Next, to determine the association between *BoLA-DRB3* and PVL in the Holstein cattle population, we distributed infected cattle into three groups according to proviral load: low PVL (LPVL; 34 ≤ PVL ≤ 85; 14 cows), moderate PVL (89 ≤ PVL ≤ 25,858; 17 cows), and high PVL (HPVL; 35,138 ≤ PVL ≤ 98,725; 14 cows), as shown in Table 3 and Figure 1. Referring to previous studies [60], the top 30% of cows with high PVL and the bottom 30% of cows with low PVL were selected as HPVL and LPVL, respectively.

### 3.4. Analysis of the Association between BoLA-DRB3 and BLV PVL in Holstein Cows

The influence of host factor *BoLA-DRB3* alleles on provirus copies in Holstein cows infected with BLV was analyzed. This analysis has not been reported before. We calculated the allelic frequencies and compared the frequency distribution of alleles in the LPVL and HPVL cows as determined by direct counting, calculation of the OR, and *p*-value based on the Chi-square test (Table 4). Alleles with OR > 1 were categorized as resistant, whereas those with OR < 1 were considered susceptible. Our present results showed that DRB3*015:01 (28.6%), DRB3*011:01 (17.9%), DRB3*012:01 (14.3%), and DRB3*001:01 (14.3%) were the most frequent alleles (>10%) in cattle with LPVL. The four most frequent alleles in the HPVL group were DRB3*015:01 (42.9%), DRB3*001:01 (21.4%), DRB3*011:01 (10.7%), and DRB3*027:03 (10.7%) (Table 4). A significant association was observed between BoLA-DRB3*012:01 and LPVL (*p* = 0.0379), as shown in Table 4. The findings represent the first description of the association of DRB3*012:01 with resistance to the BLV proviral load profile in Holsteins. In contrast, no significant association between *BoLA-DRB3* alleles and the BLV HPVL profile was found.

## 4. Discussion

This study examined the distribution of the *BoLA-DRB3* allele and the association of the BLV proviral load in Holstein cattle in Egypt. First, we identified 18 previously reported alleles for the *BoLA-DRB3* gene in the genomes of 121 Holstein cows using the PCR-SBT method. To our best knowledge, this is the first report to identify the distribution of *BoLA-DRB3* alleles in Holstein cattle in Egypt. Second, the frequency of BLV-infected versus -uninfected cattle within each *BoLA-DRB3* allele was compared. BoLA-DRB3*015:01 was identified as a susceptible allele for BLV infection in the tested Holsteins, whereas BoLA-DRB3*010:01 was considered a resistance allele for BLV infection. This is the first report to demonstrate that *BoLA-DRB3* polymorphisms affect BLV infection in Holstein cattle in Egypt. Third, our association studies between *DRB3* alleles in individuals carrying an HPVL and LPVL are the first to demonstrate the association of BLV LPVL with the BoLA-DRB3*012:01 allele in the tested Holstein cows. Thus, our results demonstrate that the *BoLA-DRB3* polymorphism confers susceptibility to BLV PVL and BLV infection in Holstein cattle in Egypt. Our results may be useful for the disease control and eradication of BLV through genetic selection.

The dairy industry and customers worldwide place growing priority on improving livestock health [61,62,63]. The dairy sector has focused on EBL because of its significance in certifying the health of animals and semen intended for export. Whether BLV poses a risk to human health is also a matter of concern [64]. Recent studies have raised the possibility that BLV is related to human breast cancer. Screening of cattle herds and milk products is encouraged to reduce the likelihood of viral transmission to humans [65]. The import of live dairy animals is essential for the effectiveness of Egypt’s dairy system [66]. According to numerous reports, BLV is spreading among cattle in Egypt, particularly in dairy farms [52,53,55,67]. Egypt has not established a national control program for BLV infection in dairy cattle, despite the fact that the import of unscreened heifers or frozen semen has brought the virus into the country [51,68,69]. Trials using preventative interventions were effective in reducing the clinical effects of BLV infection but did not completely eradicate the virus [16]. To effectively implement control measures in the dairy industry, breeds that are less susceptible or even resistant to BLV infection should be chosen based on genetic traits that are advantageous for production (e.g., milk supply, growth, and reproduction) [70,71]. The recent Kinetic Study on breed selection based on *BoLA-DRB3* allele resistance showed that it is an efficient method for lowering and controlling BLV infections in Japan [21]. Recently, it has been shown that a BLV eradication program utilizing resistant cattle as a biological barrier and preferential elimination of susceptible cattle is an effective strategy to maximally reduce BLV prevalence and PVL in stall barn dairy farms in Japan [57]. Egypt is confronted with the spread of BLV infection; however, no effective vaccines or treatment methods are available. Therefore, genetic selection based on the *BoLA-DRB3* allele may be a promising strategy to restrict BLV transmission. By using more bulls carrying resistant genetic markers for breeding or artificial insemination, a genetic selection-based control strategy will increase the pattern of inheritance and allow the expansion of its frequency. Additionally, it is likely that there will be a lot of interest in the global semen trade due to the rising demand for semen from bulls that have the *BoLA-DRB3*-resistant marker [72]. On the opposing side of the argument, other inheriting factors must be taken into account when using such a strategy. As an example, inspecting the dairy herd sire for BLAD (bovine leukocyte adhesion deficiency), an autosomal recessive congenital disease characterized by recurrent bacterial infections, fatal illness, and deaths in Holstein cattle, is necessary [73]. Therefore, the selection strategy for disease control must take genetic disorders into account.

The allelic diversity of the *BoLA-DRB3* gene in several cattle breeds in Egypt remains unknown. Analysis of the distribution of *BoLA-DRB3* alleles in Holsteins in the present study revealed a preserved pattern that is largely consistent with previously published findings in the Holstein breed [32,74,75,76]. BoLA-DRB3*015:01, *011:01, *001:01, *014:011, *009:02, *010:01, *027:03, and *012:01 were the most frequently detected alleles in Holstein cattle studied here. These *BoLA-DRB3* allelic frequencies in Holsteins were, at least in part, in line with those reported in Holsteins in Canada, which were *16 (015:01), *22 (011:01), *24 (001:01), *8 (012:01), *23 (027:03), and *11 (009:02/009:01) [36]. Additionally, in Holstein Friesians, BoLA-DRB3*001:01, *007:01, *009:02, and *020:02 were reported from cattle samples from Britain, whereas, BoLA-DRB3*006:01, *009:01, *012:01, and *017:01 were reported from American Holstein Friesians [77]. Notably, the most prevalent allele among the examined alleles was BoLA-DRB3*015:01 (20.25%). Consistent with previous studies, BoLA-DRB3*015:01 appeared to be widely distributed in Holstein cattle from various nations, including 18.2% in Bolivia, 17.7% in Paraguay, 21.4% in Peru, 21.7% in Chile, 14.7% in Argentina, 11.58% in Iran, and 27.1% in Japan [78,79,80].

To determine the effect of *BoLA-DRB3* on BLV infections in Holstein cattle in Egypt, we compared *BoLA-DRB3* allele frequencies between BLV-infected cattle (45 heads: PVL was estimated to be “≥1” by BLV-CoCoMo-qPCR-2) and BLV-non-infected cattle (76 heads: PVL was estimated to be “0” by BLV-CoCoMo-qPCR-2). DRB3*015:01 was the most frequent allele present in the infected group and was associated with susceptibility to BLV infection in the investigated Holstein cattle (OR = 0.25; *p* < 0.0001). Our results are supported by previous investigations that described the strong link of DRB3*015:01 with BLV-infected Holstein cattle with a high viral load of virus copies [29,48]. This finding is consistent with findings for Argentinean dairy Holsteins and Japanese Holsteins harboring HPVL [29,48,81]. Interestingly, BoLA-DRB3*015:01 has been linked to both the robust viral infection of white blood cells through syncytium formation and high PVL levels in the circulation of infected individuals [21]. Dams with the BLV PVL-susceptible allele DRB3*015:01 had higher levels of the BLV provirus in their milk [26]. Similarly, DRB3*015:01 has been linked to mastitis susceptibility in Japanese Holstein cattle [33]. In contrast, among the healthy group in the present study, DRB3*011:01 was the most frequent allele (17.11%); its frequency in the infected group was 16.67%, and the calculated odds were nearly equal (OR = 1.03; *p* = 1.00). Previously, DRB3*011:01 was determined to be a neutral allele (not associated with resistance or susceptibility to the BLV provirus load). In contrast, BoLA-DRB3*011:01 was associated with susceptibility to BLV infection in Vietnamese Holstein cattle [60]. However, in two previous studies, BoLA-DRB3*011:01 was associated with resistance to disease progression to the lymphoma stage in Holsteins [30,80]. In the present study, BoLA-DRB3*010:01 displayed a resistance association with BLV infections. In a previous report, BoLA-DRB3*010:01 was associated with resistance to lymphoma but not PVL [30]. Thus, the association between *BoLA-DRB3* and BLV infection profiles should be validated in future studies with a larger number of animals.

Notably, animals with high PVL serve as virus spreaders and expose other animals to genetically identical proviruses when they are in close physical contact for an extended period. Dams with high PVL promote the vertical and horizontal spread of the virus [22,24,25,27,46,51,82,83,84,85]. In PVL association studies, DRB3*015:01 and DRB3*012:01 have often been reported as susceptibility alleles to PVL. In contrast, DRB3*002:01 and DRB3*014:01:01 alleles were found to be associated with BLV resistance [29,81]. Furthermore, the DRB3*009:02 allele was most strongly associated with PL resistance or the LPVL profile in Holstein cattle [29,86]. Additionally, the risk of the vertical transmission of BLV in dams and calves with resistant alleles was previously shown to be significantly lower than that in dams and calves with susceptible alleles [46]. Here, we found that BoLA-DRB3*012:01 (*p* = 0.04) was associated with a low PVL in infected Holsteins (LPVL). BLV resistance or susceptibility may not be related only to a single allele of the *DRB3.2* gene [81]. Indeed, despite being a susceptible allele in previous reports, BoLA-DRB3*012:01 was absent in cattle with high PVL. In contrast, DRB3*009:02, DRB3*002:01, DRB3*014:01:01, and DRB3*015:01 showed no statistically significant associations with the PVL profile among the tested Holsteins here. Therefore, it is important to examine the *BoLA-DRB3* polymorphism in more detail in various cattle breeds in Egypt. Further research involving a large number of BLV-positive samples is required to gain a thorough understanding of the relationship between *BoLA-DRB3* and the BLV infection profile in Holstein populations in Egypt. In our future plan, we think that a thorough investigation of BLV infection and PVL in Holsteins across different Egyptian provinces as well as a review of additional risk factors could help to clarify the epidemiology of the disease.

In addition to the association study, further studies are indispensable to clarify why the *BoLA-DRB3* polymorphism is associated with resistance and susceptibility to BLV PVL, as follows. Functional BoLA class II DR molecules are heterodimeric transmembrane glycoproteins, which are formed by the non-covalent association between α- and β-chains, encoded by distinct genes, a single polymorphic gene, *BoLA-DRA*, and a highly polymorphic gene, *BoLA-DRB3*, respectively. The α1 and β1 domains form the peptide binding grooves, 1, 4, 6, 7, and 9. Thus, the peptide-binding preference of the BoLA class II DR molecule is critically governed by the polymorphisms of the BoLA-DRβ chain. The BoLA-DR molecules are expressed by specific immunocompetent cells, such as B cells, macrophages, dendritic cells, and activated T cells, and they present peptides derived from exogenous antigens to CD4^+^ T cells and initiate the immune response. Indeed, experimental infection in an ovine BLV model showed that amino acid positions 70 and 71 within the β1 domains in the peptide-binding groove 4 of ovine leukocyte antigen-DRβ were critical for the resistance or susceptibility of BLV-induced lymphoma by affecting the efficiency of Th1 activation [87]. This result highlights the importance of amino acid and structure analysis of BoLA-DRβ in understanding how BoLA-DRβ polymorphisms affect lymphoma susceptibility. Furthermore, structure and electrostatic charge modeling indicated that binding pocket 9 of susceptible DRB*3* was neutrally charged, while resistant alleles were positively charged [88]. These findings suggest that cattle with a particular allele could bind with different peptides and then induce differential immune responses. However, further in vitro experiments that identify antigenic peptides directly within the binding cleft of the BoLA-DR molecule related to resistance or susceptibility are essential. In addition, in vivo experimental infection with BLV using cattle carrying susceptibility or resistant alleles for BLV infection and PVL is indispensable.

## 5. Conclusions

In this study, we showed for the first time that BLV PVL and BLV infections are linked to different *BoLA-DRB3* alleles in Holstein cattle in Egypt. BoLA-DRB3*010:01 and BoLA-DRB3*015:01 are strongly associated with resistance and susceptibility to BLV infection, respectively, in Holsteins in Egypt, and BoLA-DRB3*012:01 is directly linked to the ability to carry low levels of PVL in their circulation.

## Figures and Tables

**Table 1:** *BoLA-DRB3* allele frequencies in Holstein cattle in Egypt.

*BoLA-DRB3*	Holsteins in Egypt *n* ^a^ = 121		
Allele	Frequency	CI ^b^ (95%)	
* 001:01	15.70 ^c^	11.48	21.04
* 002:01	2.89	1.27	6.12
* 005:01	0.41	0.02	2.64
* 006:01	0.83	0.14	3.28
* 007:01	1.65	0.53	4.46
* 007:04	0.83	0.14	3.28
* 008:01	0.83	0.14	3.28
* 009:02	7.44	4.59	11.69
* 010:01	7.44	4.59	11.69
* 011:01	16.94	12.56	22.40
* 012:01	6.61	3.95	10.72
* 013:01	0.41	0.02	2.64
* 014:01:01	8.68	5.58	13.14
* 015:01	20.25	15.48	25.98
* 016:01	1.24	0.32	3.88
* 017:01	0.83	0.14	3.28
* 018:01	0.83	0.14	3.28
* 027:03	6.20	3.63	10.22

^a^ *n*: Number of tested animals. ^b^ CI: Confidence interval of 95%. ^c^ The most frequent alleles are underlined (greater than 5%).

**Table 2:** Association of *BoLA-DRB3* alleles with BLV-non-infected and -infected Holstein cattle in Egypt.

*BoLA-DRB3* Alleles	Holstein Cattle				Fisher’s Exact Test ^b^		Susceptibility
	BLV-Non-Infected Cattle *n* ^a^ = 76		BLV-Infected Cattle *n* = 45		OR ^c^	*p*-Value	
	Count	(%)	Count	(%)			
* 001:01	23	(15.13)	15	(16.67)	0.89	0.86	-
* 002:01	5	(3.29)	2	(2.22)	1.50	1.00	-
* 005:01	1	(0.66)	0	(0.00)	-	1.00	-
* 006:01	1	(0.66)	1	(1.11)	0.59	1.00	-
* 007:01	4	(2.63)	0	(0.00)	-	0.30	-
* 007:04	0	(0.00)	2	(2.22)	0.00	0.14	-
* 008:01	2	(1.32)	0	(0.00)	-	0.53	-
* 009:02	14	(9.21)	4	(4.44)	2.18	0.21	-
* 010:01	18	(11.84)	0	(0.00)	-	0.00	R ^d^
* 011:01	26	(17.11)	15	(16.67)	1.03	1.00	-
* 012:01	10	(6.58)	6	(6.67)	0.99	1.00	-
* 013:01	1	(0.66)	0	(0.00)	-	1.00	-
* 014:01:01	15	(9.87)	6	(6.67)	1.53	0.48	-
* 015:01	18	(11.84)	31	(34.44)	0.26	0.00	S
* 016:01	3	(1.97)	0	(0.00)	-	0.30	-
* 017:01	1	(0.66)	1	(1.11)	0.59	1.00	-
* 018:01	1	(0.66)	1	(1.11)	0.59	1.00	-
* 027:03	9	(5.92)	6	(6.67)	0.88	0.79	-

^a^ *n*: Number of tested animals. ^b^ Association between *BoLA-DRB3* allele and BLV infection was ascertained using Fisher’s exact test by comparing the rate of the *BoLA-DRB3* allele between BLV-uninfected and -infected cattle. ^c^ OR: odds ratio, ^d^ R: resistant allele, S: susceptible allele.

**Table 3:** Summary of CoCoMo-qPCR-based proviral load (PVL) determination for the BLV and classification criteria for PVL.

*n* ^a^ of Tested Animals (Heads)	*n* of qPCR-Positive Samples (Heads)	Mean PVL ^b^ (Copies/10^5^ Cells)	PVL Category	PVL Range (Copies/10^5^ Cells)	*n* of Cattle (Heads)
121	45	20,027	Low PVL (LPVL)	34–85	14
			Moderate	89–25,858	17
			High PVL (HPVL)	35,138–98,725	14

^a^ *n*: Number. ^b^ PVL: Proviral load. Quantification of BLV PVL was determined by BLV-CoCoMo-qPCR-2 methods (Nippon Gene).

**Table 4:** Association between *BoLA-DRB3* alleles and BLV proviral load in Holstein cattle in Egypt.

DRB3 Allele	Holstein Cattle BLV (+)				Chi-Square Test ^b^		Susceptibility
	LPVL (*n* ^a^ = 14)		HPVL (*n* ^a^ = 14)				
	Count	Freq.	Count	Freq.	OR ^c^	*p*-Value	
* 001:01	4	14.3	6	21.4	0.6111	0.4853	
* 002:01	0	0	1	3.6	0	0.3130	
* 006:04	0	0	1	3.6	0	0.3130	
* 007:04	1	3.6	0	0	-	0.3130	
* 009:02	2	7.1	0	0	-	0.1498	
* 011:01	5	17.9	3	10.7	1.8116	0.4450	
* 012:01	4	14.3	0	0	-	0.0379	R ^d^
* 014:01:01	2	7.3	1	3.6	2.0769	0.5529	
* 015:01	8	28.6	12	42.9	0.5333	0.2646	
*017:01	0	0	1	3.6	0	0.3130	
* 018:01	0	0	0	0	-	-	
* 027:03	2	7.1	3	10.7	0.6410	0.6393	

^a^ *n*: Number of tested animals. ^b^ Association between BoLA-DRB3 allele and BLV proviral load profile was ascertained using Chi-square test by comparing the frequency distribution of alleles between low proviral load-harboring cows (LPVL) and high proviral load-harboring cows (HPVL). ^c^ OR: Odds ratio. ^d^ R: Resistant allele.

## Data Availability

The data presented in this study are available on request from the corresponding author.

## Supplementary Materials

The following supporting information can be downloaded at https://www.mdpi.com/article/10.3390/pathogens12121451/s1, Table S1: General information about dairy cattle population analyzed in this study; Table S2: Sequences of the used primer/probe in this study.
