# Supplementary material for: BoLA-DRB3 Polymorphism Associated with Bovine Leukemia Virus Infection and Proviral Load in Holstein Cattle in Egypt
Source: Pathogens. 2023 Dec 14;12(12):1451. doi: 10.3390/pathogens12121451 (PMC10746042; doi:10.3390/pathogens12121451)
Supplement: Supplementary file 1 [file pathogens-12-01451-s001.zip › Table S2.pdf]

Table S2. Sequences of the used primer/probe in this study.

Target	Primer/probe ID	Primer sequence 5′-3′	Reference
1) BLV-PVL-COCOMO- qPCR-2			
BLV-LTR	CoCoMo-FRW	AATCCMNMYCYKDAGCTGCTGAYYTCACCT	Takeshima et al. (53)
		ATCCACACCCTGAGCTGCTGCACCTCACCT	Takeshima et al. (53)
		primers mixture 1:10	
	CoCoMo-REV	TTGCCTTACCTGMCSSCTKSCGGATAGCCGA	Takeshima et al. (53)
	FAM-LTR probe	FAM-CTCAGCTCTCGGTCC-NFQ-MGB	Jimba et al. (15)
BoLA-DRA	DRA-FW	CCCAGAGTATGAAGCTCCAGCCC	Takeshima et al. (53)
	DRA-RW	CCCTCGGCGTTCAACGGTGT	Takeshima et al. (53)
	FAM-DRA probe	FAM-TGTGTGCCCTGGGC -NFQ-MGB	Takeshima et al. (53)
2) BoLA-DRB3 Genotyping			
BoLA-DRB3	DRB3FRW	CGCTCCTGTGAYCAGATCTATCC	Takeshima et al. (55)
	DRB3REV	CACCCCCGCGCTCACC	Takeshima et al. (55)
